# Serum Axl predicts histology-based response to induction therapy and long-term renal outcome in lupus nephritis

**DOI:** 10.1371/journal.pone.0212068

**Published:** 2019-02-11

**Authors:** Ioannis Parodis, Huihua Ding, Agneta Zickert, Guillaume Cosson, Madiha Fathima, Caroline Grönwall, Chandra Mohan, Iva Gunnarsson

**Affiliations:** 1 Division of Rheumatology, Department of Medicine, Karolinska Institutet, Stockholm, Sweden; 2 Rheumatology, Karolinska University Hospital, Stockholm, Sweden; 3 Department of Biomedical Engineering, University of Houston, Houston, Texas, United States of America; 4 Department of Rheumatology, Renji Hospital, Shanghai JiaoTong University School of Medicine, Shanghai, China; Instituto Nacional de Ciencias Medicas y Nutricion Salvador Zubiran, MEXICO

## Abstract

Axl is a receptor tyrosine kinase with important functions in immune regulation. We investigated serum levels of soluble (s)Axl in lupus nephritis (LN) in association with renal disease activity, tissue damage and treatment response. We surveyed 52 patients with International Society of Nephrology/Renal Pathology Society (ISN/RPS) class III/IV LN and 20 healthy controls. Renal biopsies were performed at the time of active LN and post-treatment. Patients were classified as clinical responders (CRs) or clinical non-responders based on the American College of Rheumatology (ACR) criteria. Improvement by ≥50% in renal activity index scores defined histological responders (HRs). sAxl levels were elevated in patients compared to controls (median: 18.9 ng/mL), both at baseline (median: 45.7; *P*<0.001) and post-treatment (median: 41.2 ng/mL; *P*<0.001). Baseline sAxl levels were higher in patients with class IV (median: 47.7 ng/mL) versus class III (median: 37.5 ng/mL) nephritis (*P* = 0.008), and showed moderate correlations with albuminuria (r = 0.30, *P* = 0.030) and creatinine (r = 0.35, *P* = 0.010). Baseline sAxl levels decreased in CRs (*P* = 0.002) and HRs (*P*<0.001), but not in non-responders; levels ≥36.6 ng/mL yielded a >5 times higher probability of histology-based response (odds ratio, OR: 5.5; 95% confidence interval, CI: 1.2–25.1). High post-treatment sAxl levels were associated with worsening in chronicity index scores (*P* = 0.025); low levels predicted favourable renal outcome (creatinine ≤88.4 μmol/L) 10 years after the baseline renal biopsy (area under the curve: 0.71; 95% CI: 0.54–0.89). In conclusion, sAxl may prove useful as a marker of renal activity, histological response to immunosuppression, and renal damage progression in LN. Persistently high sAxl levels after completion of treatment may be indicative of a need for treatment intensification.

## Introduction

Lupus nephritis (LN) is a severe manifestation in systemic lupus erythematosus (SLE). Despite improvements in therapeutic management, LN remains a major cause of morbidity in SLE [1]. The current gold standard for the diagnosis and classification of LN is the renal biopsy, and repeat biopsies have been proven important in determining treatment outcomes [2–6]. Given the potential risks of this procedure, it is crucial to identify biomarkers for tracking renal activity and predicting response to therapy and long-term prognosis.

Axl belongs to a receptor tyrosine kinase subgroup consisting of three members (Tyro3, Axl and Mer, collectively designated as TAM), and is expressed in several cell types, in particular in myeloid cells [7]. The TAM receptors are important in homeostasis, including the regulation of innate immune responses, and play key roles in apoptotic cell clearance [8–10]. Impaired clearance of apoptotic material has been hypothesised to play a pivotal role in the pathogenesis of SLE [11], and investigation of the TAM pathways in SLE is therefore gaining increasing interest.

Notably, TAM triple mutant mice develop a severe lymphoproliferative phenotype with clinical features of SLE and rheumatoid arthritis, accompanied by production of autoantibodies [12]. However, the Axl receptor and its ligand Gas6 have also been linked to cell proliferation and survival with oncogenic properties [13]. Of particular relevance for lupus nephritis, Gas6 has been reported to be an autocrine growth factor for mesangial and epithelial cells, and the Axl pathway has been suggested to contribute to nephritis through its essential role in renal mesangial cell proliferation [14–17]. In line with the autoimmune profile in knockout (KO) mice, Axl signalling has also been demonstrated to downregulate inflammation and suppress type I interferon signalling through induction of SOCS1/3 [10] and the transcriptional inhibitor Twist in innate immune cells, leading to a subsequent suppression of inflammatory cytokine expression [18]. Interestingly, the extracellular domain of Axl and other TAM receptors may be shed from the cell surface by proteases to generate soluble receptors, in a process that could play a role in the *in vivo* regulation of these receptors and may be important in SLE pathogenesis [19, 20].

In clinical surveys, soluble (s)Axl levels have been found to be elevated in patients with SLE compared to healthy subjects while membrane Axl expression on monocytes and macrophages was decreased [21]. Furthermore, plasma concentrations of sAxl have been demonstrated to correlate with SLE activity [22]. In a recent biomarker screening study of Caucasian, Hispanic and African American SLE patients [23] as well as a subsequent validation study of Chinese patients [24], serum levels of sAxl could discriminate active renal from active non-renal SLE, supporting the notion that Axl might be a molecule of particular interest in LN.

In order to further clarify the role of Axl in renal SLE, we assessed serum levels of sAxl in a longitudinal cohort of LN patients, with renal biopsies performed on the occasion of an active LN and after completion of induction treatment. We sought to investigate potential associations of serum sAxl with renal disease activity, renal damage, and response to treatment. Finally, we investigated the performance of serum sAxl as a predictor of the long-term renal outcome.

## Materials and methods

### Patients

Patients from the Karolinska University Hospital have been enrolled in a prospective cohort study on the occasion of an active biopsy-proven LN from 1996 onwards. Patients in this cohort study are followed longitudinally, and clinical and laboratory data are collected prospectively. In the present retrospective analysis, we included 52 patients from this cohort enrolled between 1996 and 2011 with LN class III (n = 20) or IV (n = 32) based on the 2003 International Society of Nephrology/Renal Pathology Society (ISN/RPS) classification [25]; all renal biopsies were reassessed and evaluated according to the 2003 ISN/RPS classification system by the same renal pathologist at the Karolinska University Hospital. All patients fulfilled the 1982 revised American College of Rheumatology (ACR) criteria [26] and the 2012 Systemic Lupus International Collaborating Clinics (SLICC) criteria [27] for classification of SLE. Demographics and baseline clinical data are presented in Table 1.

**Table 1:** Baseline characteristics.

	ISN/RPS class III/IV N = 52	ISN/RPS class V N = 12
**Age** (years); M (IQR)	30.0 (23.9–39.3)	45.7 (28.3–57.1)
**Women**; n (%)	45 (86.5)	10 (83.3)
**Ethnicity**		
Caucasian; n (%)	50 (96.2)	10 (83.3)
African/African American; n (%)	1 (1.9)	0 (0.0)
Asian; n (%)	1 (1.9)	2 (16.7)
**SLE disease duration** (years); M (IQR)	2.6 (0.1–7.6)	9.5 (0.3–20.4)
**Time between evaluations** (months); M (IQR)	7.6 (6.6–8.5)	8.6 (7.7–10.3)
**SLEDAI-2K**; M (IQR)	16.0 (12.0–20.0)	11.5 (10.0–18.3)
**Prednisone equivalent dose** (mg/day); M (IQR)	10.0 (0.0–20.0)	5.6 (0.0–15.0)
**Antimalarials**; n (%)	15 (28.8)	2 (16.7)
**Immunosuppressive drugs**; n (%)	22 (34.4)	3 (25.0)
Azathioprine; n (%)	12 (23.1)	2 (16.7)
Methotrexate; n (%)	4 (7.7)	0
Mycophenolate mofetil; n (%)	2 (3.8)	1(8.3)
Oral cyclophosphamide; n (%)	1 (1.9)	0
None; n (%)	33 (63.5)	9 (75.5)
**ACE inhibitors and/or ARBs**; n (%)	38 (73.1)	12 (100)
**Comorbid hypertension**; n (%)	25 (49.0), N = 51	9 (75.0)
**Comorbid diabetes**; n (%)	5 (9.8), N = 51	2 (16.7)

The number of observations was 52 in the ISN/RPS class III/IV group and 12 in the class V group unless otherwise indicated.

ISN/RPS, International Society of Nephrology/Renal Pathology Society; SLE, systemic lupus erythematosus; SLEDAI-2K, SLE Disease Activity Index 2000; ACE, angiotensin-converting enzyme; ARBs, angiotensin receptor blockers; M, median; IQR, interquartile range.

Additionally, we recruited 20 healthy controls of similar age (median: 32.0 years; interquartile range, IQR: 25.5–40.8 years; *P* = 0.425 derived from Mann-Whitney *U* test) and sex (85.0% women; *P* = 1.000 derived from Fisher’s Exact test) distribution for the purpose of comparisons. Of those 20 healthy individuals, 12 were Caucasian, four were African and four were Asian. Finally, we conducted a pilot experiment in a limited cohort of twelve patients with pure membranous lupus nephritis (ISN/RPS class V). The patients were enrolled from the same prospective LN cohort as the proliferative LN patients, and during the same study period. The renal biopsies (twelve biopsies at baseline and eleven biopsies post-treatment) were evaluated by the same renal pathologist. Baseline characteristics of this cohort are also presented in Table 1.

After confirmation of LN class III/IV, the patients received induction therapy with cyclophosphamide (n = 40), mycophenolate mofetil (n = 9) or rituximab (n = 3), combined with glucocorticoids. The choice of treatment was directed by the treating physician. Cyclophosphamide was administered as monthly intravenous pulses of 0.5–1 g according to the modified National Institutes of Health (NIH) protocol [28], or as infusions of 0.5 g every second week for a total of three months (six infusions) according to the low-dose Euro-Lupus Nephritis Trial (ELNT) cyclophosphamide protocol [29]. The administration of mycophenolate mofetil was oral and comprised daily doses ranging from 2 to 3 g, based on individual decisions by the treating physician. Rituximab was given in combination with cyclophosphamide [30], or as intravenous infusions of 1 g at week 0 and 2 in cases where cyclophosphamide was considered inappropriate.

For evaluation of the treatment response, a repeat renal biopsy was performed after completion of induction treatment in addition to clinical evaluation and blood and urine tests. The time between baseline and post-treatment evaluation ranged from 5.0 to 15.6 months (median: 7.6 months), depending on the induction treatment regimen and the clinical course. In the majority of the cases, the post-treatment evaluation was conducted 6–12 months after the baseline renal biopsy (n = 46, 88.5%); in three patients, the post-treatment assessment was conducted earlier than 6 months, and in three patients later than 12 months from baseline.

The study was conducted in accordance with the ethical principles of the declaration of Helsinki, and the study protocol was reviewed and approved by the regional ethics review board, i.e. the Ethics Council at the Karolinska Institutet in Stockholm. Written informed consent was obtained prior to enrolment from all adult individuals participating in the study, and also from the next of kin, caretakers, or guardians on behalf of the minors or children enrolled.

### Assessment of serum sAxl levels

Serum from patients was collected at the time of baseline and post-treatment renal biopsies, and from controls at the time of enrolment. The serum samples were cryopreserved at -80°C until the analysis. Serum levels of sAxl were determined using enzyme-linked immunosorbent assay (ELISA) (R&D Systems, Inc., Minneapolis, Minnesota, USA; catalogue number: DY154). Serum samples were diluted 1:50. All assays were undertaken according to the manufacturer's protocol. Optical density at a wavelength of 450 nm was measured using an ELx808^TM^ Absorbance Microplate Reader (BioTek Instruments, Inc., Winooski, Vermont, USA), and the concentrations of the samples were calculated using a standard curve. All samples were analysed in duplicate and all experiments were performed in a blinded manner.

### Determination of autoantibody levels

Serum levels of IgG antibodies to double-stranded DNA (anti-dsDNA; reference: <5 IU/mL) were measured using multiplex immunoassay technology (BioPlex 2200 System, Bio-Rad Laboratories, Inc., Hercules, California, USA). Levels of IgG antibodies to complement component 1q (anti-C1q; reference: <14 U/mL) were determined using ELISA (Alegria, ORGENTEC Diagnostika GmbH, Germany). Complement C3 and C4 levels were determined using nephelometry.

### Definitions and surveillance methods

The 2003 ISN/RPS classification [25] was used to define the LN subsets. The renal biopsies were additionally scored using the renal activity and chronicity indices [31]. Urine test strips and urinary sediment were examined on each biopsy occasion. Proteinuria was estimated by the 24-hour (h) urine (U)-albumin excretion or U-albumin/creatinine ratio. Global disease activity was assessed using the SLE Disease Activity Index 2000 (SLEDAI-2K) [32].

Renal function was assessed on both biopsy occasions and ten years after the baseline biopsy using the plasma creatinine concentration (μmol/L) and the creatinine-based estimated glomerular filtration rate (eGFR), calculated with the revised Lund-Malmö equation (LM eGFR) [33]. For patients on dialysis, we considered the last creatinine value prior to initiation of dialysis. In accordance with the National Kidney Disease Education Program recommendations, we supressed creatinine values calculated before the method shift to isotope dilution mass spectrometry with a factor 175/186.

### Definitions of clinical and histological response to treatment

After completion of induction treatment, the patients were classified into clinical responders (CRs) if they showed ≥50% reduction in proteinuria to levels ≤2 g/day, normal or improved by ≥25% eGFR and inactive urinary sediment, as suggested by the ACR criteria [34], or clinical non-responders (CnRs) if they did not. Additionally, patients showing ≥50% improvement in renal activity index scores in post-treatment compared to baseline biopsies were considered histological responders (HRs); all other patients were considered histological non-responders (HnRs) [35].

### Definition of good long-term renal outcome

To conform with the Euro-Lupus Nephritis Trial (ELNT), we considered creatinine concentrations of ≤88.4 μmol/L a good renal outcome [36, 37]. We applied this definition at the 10-year follow-up.

### Statistics

We used the non-parametric Wilcoxon signed-rank test for pairwise comparisons between baseline and post-treatment measurements and the Mann-Whitney *U* test for comparisons between unrelated samples, *e*.*g*. between LN patients and healthy controls. For comparisons between binomial variables, we used the Pearson’s chi-square (*χ*^2^) or the Fisher’s exact test as appropriate.

For correlation analyses, we used the Spearman’s rank correlation coefficient. Logistic regression was used for further evaluation of sAxl as a predictor of treatment outcomes and long-term prognosis as appropriate. Receiver operating characteristic (ROC) curves were constructed for illustrative purposes, and coordinate points were examined to determine optimal threshold values. Data from the assessment of autoantibody levels were bounded by the detection limits of the assays; censored values were set to half the lower or twice the upper detection limit. *P*-values <0.05 were considered statistically significant.

Statistical analyses were performed using the IBM SPSS Statistics 25 software (IBM Corp., Armonk, New York, USA).

## Results

According to renal pathology in baseline biopsies, 20 patients had an ISN/RPS class III lupus nephritis with or without a concomitant membranous pattern (±V) and 32 patients had an ISN/RPS class IV (±V) glomerulonephritis (Table 2). A total of ten patients had a concomitant membranous pattern (ISN/RPS class V) (Table 3); the other 42 patients had a pure proliferative LN.

**Table 2:** Comparisons between baseline and post-treatment evaluation.

	Baseline	Post-treatment	*P*-value
**ISN/RPS class**; n (%)			
I	0	1 (1.9)	-
II	0	15 (28.8)	-
III^a^	20 (38.5)	17 (53.1)	-
IV^a^	32 (61.5)	9 (17.3)	-
V	0	9 (17.3)	-
Glomerular vasculitis	0	1 (1.9)	-
**Activity Index**; M (IQR)	6.0 (5.0–8.0)	2.0 (1.0–3.0)	**<0.001**
**Chronicity Index**; M (IQR)	1.0 (0.0–2.0)	2.0 (1.0–3.0)	**<0.001**
**SLEDAI-2K**; M (IQR)	16 (12.0–20.0)	4.0 (2.0–7.8)	**<0.001**
**sAxl levels** (ng/mL); M (IQR)	45.7 (36.6–56.0)	41.2 (30.8–53.9)	**0.001**
**Anti-dsDNA** (positive); n (%)	52 (100)	48 (92.3)	-
**Anti-dsDNA** (IU/mL); M (IQR)	200.0 (50.0–600^c^), N = 51	26.0 (13.5–74.5), N = 49	**<0.001**
**Anti-C1q** (positive); n (%)	38 (74.5), N = 51	26 (50.0)	-
**Anti-C1q** (U/mL); M (IQR)	45.4 (13.4–78.4), N = 51	13.7 (6.8–36.2)	**<0.001**
**C3** (g/L); M (IQR)	0.50 (0.36–0.73)	0.80 (0.62–1.08)	**<0.001**
**C4** (g/L); M (IQR)	0.08 (0.04–0.13)	0.13 (0.10–0.17)	**<0.001**
**24-h U-albumin** (g/day); M (IQR)	1.4 (0.7–2.6), N = 51	0.2 (0.04–0.8), N = 51	**<0.001**
**Creatinine** (μmol/L); M (IQR)	76.5 (62.5–91.3)	72.5 (61.4–81.9)	**0.028**
ISN/RPS class III^b^	66.8 (57.0–79.0), N = 20	68.0 (55.8–80.7), N = 20	0.936
ISN/RPS class IV^b^	82.9 (68.0–100.2), N = 32	73.4 (63.7–83.7), N = 32	**0.004**
**eGFR** (mL/min/1.73 m^2^); M (IQR)	80.0 (71.3–97.0)	87.5 (77.0–97.8)	**0.022**
ISN/RPS class III^b^	89.0 (79.3–103.0), N = 20	89.0 (81.0–98.5), N = 20	0.881
ISN/RPS class IV^b^	75.5 (62.8–89.5), N = 32	87.0 (75.5–96.8), N = 32	**0.002**
**CKD stage**			-
Stage 1; n (%)	18 (34.6)	24 (46.2)	-
Stage 2; n (%)	25 (48.1)	24 (46.2)	-
Stage 3; n (%)	8 (15.4)	3 (5.8)	-
Stage 4; n (%)	1 (1.9)	1 (1.9)	-

The number of observations (N) was 52 unless otherwise stated. The lower and upper detection limits of the assay used for the determination of anti-dsDNA levels were 5 IU/mL and 300 IU/mL, respectively. The upper limit of the assay used for estimating anti-C1q levels was 100 U/mL. The non-parametric Wilcoxon signed rank test was used for the comparisons. Statistically significant *P*-values are in bold.

ISN/RPS, International Society of Nephrology/Renal Pathology Society; SLE, systemic lupus erythematosus; SLEDAI-2K, SLE Disease Activity Index 2000; anti-dsDNA, antibodies against double-stranded DNA; anti-C1q, antibodies against complement component 1q; eGFR, estimated glomerular filtration rate; CKD, chronic kidney disease; h, hour; U, urine; M, median; IQR, interquartile range.

^a^ Patients with ISN/RPS class III lupus nephritis were further stratified into patients with active lesions in the biopsy (class III A; n = 13 at baseline; n = 0 post-treatment), active lesions and chronic changes (class III A/C; n = 7 at baseline; n = 9 post-treatment), and chronic changes only (class III C; n = 0 at baseline; n = 8 post-treatment). Accordingly, patients with ISN/RPS class IV glomerulonephritis (segmental or global) were further stratified into patients with active lesions in the renal biopsy (class IV A; n = 16 at baseline; n = 2 post-treatment), active lesions and chronic changes (class IV A/C; n = 16 at baseline; n = 5 post-treatment), and chronic changes only (class IV C; n = 0 at baseline; n = 2 post-treatment).

^b^ Based on the baseline renal biopsy evaluation.

^c^ >300 IU/mL (upper detection limit of the assay).

**Table 3:** Comparative analysis of ISN/RPS class III versus class IV nephritis.

		ISN/RPS class III N = 20	ISN/RPS class IV N = 32	*P*-value
**Concomitant class V pattern**; n (%)		5 (25.0)	5 (15.6)	0.480^b^
**Activity Index**; M (IQR)	**Baseline**	5.0 (4.3–7.0)	6.0 (5.0–9.8)	0.165
	**Post-treatment**	1.0 (1.0–4.8)	2.0 (1.0–2.0)	0.430
**Chronicity Index**; M (IQR)	**Baseline**	0.5 (0.0–1.0)	1.0 (0.0–3.0)	0.162
	**Post-treatment**	1.0 (1.0–2.0)	2.0 (1.0–3.8)	0.084
**SLEDAI-2K** (IU/mL); M (IQR)	**Baseline**	14.5 (11.3–18.0)	16.0 (13.0–21.8)	0.146
	**Post-treatment**	4.0 (2.5–8.0)	4.0 (2.0–6.0)	0.609
**24-h U-albumin** (g/day); M (IQR)	**Baseline**	0.9 (0.3–1.4)	1.9 (1.0–3.9), N = 31	**0.003**
	**Post-treatment**	0.3 (0.03–0.7)	0.2 (0.06–1.0), N = 31	0.757
**Creatinine** (μmol/L); M (IQR)	**Baseline**	66.8 (57.0–79.0)	82.9 (68.0–100.2)	**0.011**
	**Post-treatment**	68.0 (55.8–80.7)	73.4 (63.7–83.7)	0.314
**eGFR** (mL/min/1.73 m^2^); M (IQR)	**Baseline**	89.0 (79.3–103.0)	75.5 (62.8–89.5)	**0.010**
	**Post-treatment**	89.0 (81.0–98.5)	87.0 (75.5–96.8)	0.440
**sAxl levels** (ng/mL); M (IQR)	**Baseline**	37.5 (31.4–49.0)	47.7 (40.5–62.7)	**0.008**
	**Post-treatment**	36.4 (28.3–45.6)	43.9 (30.8–54.6)	0.267
**anti-dsDNA** (IU/mL); M (IQR)	**Baseline**	200.0 (98.8–600^a^)	200.0 (27.0–600^a^), N = 31	0.373
	**Post-treatment**	22.0 (12.8–78.0), N = 18	26.0 (15.0–74.0), N = 31	0.507
**Anti-C1q** (U/mL); M (IQR)	**Baseline**	54.3 (16.0–173.8)	25.9 (12.3–71.1), N = 31	0.309
	**Post-treatment**	20.5 (6.0–38.4)	10.8 (8.2–34.5)	0.481
**C3** (g/L); M (IQR)	**Baseline**	0.49 (0.33–0.65)	0.51 (0.38–0.75)	0.469
	**Post-treatment**	0.67 (0.51–0.95)	0.82 (0.66–1.14)	0.071
**C4** (g/L); M (IQR)	**Baseline**	0.07 (0.04–0.13)	0.10 (0.04–0.13)	0.546
	**Post-treatment**	0.11 (0.07–0.16)	0.15 (0.11–0.18)	0.084
**Comorbid hypertension**; n (%)		6 (30.0)	19 (61.3), N = 31	**0.029**^c^
**Comorbid diabetes**; n (%)		2 (10.0)	3 (9.7), N = 31	1.000^b^

The number of observations (N) was 20 for the ISN/RPS class III LN group and 32 for the class IV LN group unless otherwise stated. The lower and upper detection limits of the assay used for the determination of anti-dsDNA levels were 5 IU/mL and 300 IU/mL, respectively. The upper limit of the assay used for estimating anti-C1q levels was 100 U/mL. The non-parametric Mann-Whitney *U* test was used for the comparisons unless otherwise stated. Statistically significant *P*-values are in bold.

ISN/RPS, International Society of Nephrology/Renal Pathology Society; SLE, systemic lupus erythematosus; SLEDAI-2K, SLE Disease Activity Index 2000; anti-dsDNA, antibodies against double-stranded DNA; anti-C1q, antibodies against complement component 1q; eGFR, estimated glomerular filtration rate; h, hour; U, urine; M, median; IQR, interquartile range.

^a^ >300 IU/mL (upper detection limit of the assay).

^b^ *P*-value derived from Fisher’s exact test.

^c^ *P*-value derived from Pearson’s chi-square test.

Patients with ISN/RPS class III lupus nephritis were further stratified into patients with active lesions in the biopsy (class III A; n = 13 at baseline; n = 0 post-treatment), active lesions and chronic changes (class III A/C; n = 7 at baseline; n = 9 post-treatment), and chronic changes only (class III C; n = 0 at baseline; n = 8 post-treatment). Accordingly, patients with ISN/RPS class IV glomerulonephritis (segmental or global) were further stratified into patients with active lesions in the renal biopsy (class IV A; n = 16 at baseline; n = 2 post-treatment), active lesions and chronic changes (class IV A/C; n = 16 at baseline; n = 5 post-treatment), and chronic changes only (class IV C; n = 0 at baseline; n = 2 post-treatment). A comparative analysis of patients with ISN/RPS class III versus class IV nephritis according to the baseline biopsy evaluation is presented in Table 3.

Post-treatment, most of class III/IV nephritides had reverted to class II. The renal activity index scores showed a significant decrease from a median of 6.0 to a median of 2.0 (*P*<0.001), and eGFR values improved from a median of 80.0 to a median of 87.5 mL/min/1.73 m^2^ (*P* = 0.022) (Table 2).

### Serum sAxl as a biomarker of renal disease activity and renal damage

**Fig 1:**
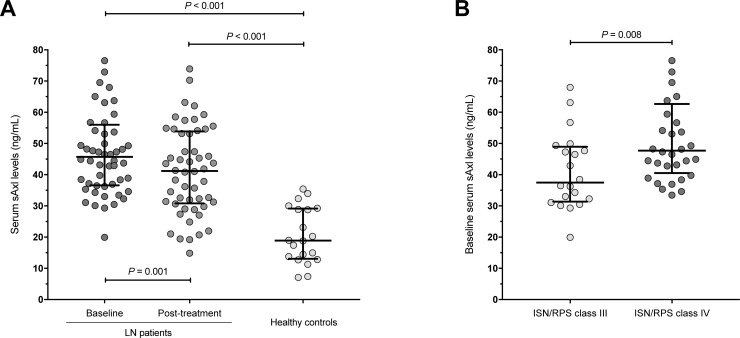
sAxl levels in LN patients and healthy subjects. The scatterplots in panel A delineate serum levels of soluble (s)Axl receptor in patients with lupus nephritis (LN) before and after induction treatment (n = 52), and in healthy controls (n = 20). Baseline serum levels of sAxl in LN patients were significantly elevated (median; IQR: 45.7; 36.6–56.0 ng/mL) compared with the healthy controls (*P*<0.001; Mann-Whitney *U* test). Following treatment, serum levels of sAxl showed a significant decrease (*P* = 0.001; Wilcoxon signed-rank test), but remained significantly higher compared to serum sAxl levels in healthy controls (*P*<0.001; Mann-Whitney *U* test). In panel B, the scatterplots delineate serum levels of sAxl across nephritis classes. Baseline serum levels of sAxl were higher in patients with ISN/RPS class IV LN compared to patients with ISN/RPS class III nephritis (*P*-value derived from Mann-Whitney *U* test). Lines and whiskers denote medians and the 25^th^ and 75^th^ percentiles. Outliers may have been omitted due to scaling.

Baseline serum levels of sAxl were significantly elevated in class III/IV LN patients (median; IQR: 45.7; 36.6–56.0 ng/mL) compared to the healthy controls (median; IQR: 18.9; 13.1–29.1 ng/mL) (*P*<0.001) (Fig 1). Following treatment, serum levels of sAxl had decreased to a median of 41.2 ng/mL (IQR: 30.8–53.9 ng/mL) (*P* = 0.001) (Table 2), but remained significantly higher compared to serum sAxl levels in healthy controls (*P*<0.001) (Fig 1). Interestingly, only patients with ISN/RPS class IV (±V) LN in baseline biopsies accounted for this decrease (*P* = 0.001), whereas sAxl levels remained stable in patients with class III (±V) nephritis (*P* = 0.332). Baseline serum levels of sAxl were higher in patients with class IV (±V) LN (median; IQR: 47.7; 40.5–62.7 ng/mL) compared to patients with class III (±V) nephritis (median; IQR: 37.5; 31.4–49.0 ng/mL) (*P* = 0.008) (Fig 1), as were creatinine (median; IQR: 82.9; 68.0–100.2 versus 66.8; 57.0–79.0 μmol/L; *P* = 0.011) and albuminuria (median; IQR: 1.9; 1.0–3.9 versus 0.9; 0.3–1.4 g/day; *P* = 0.003) levels. Changes in sAxl levels expressed as ΔsAxl did not show any correlation with changes in creatinine concentrations (r = 0.12, *P* = 0.398), or creatinine concentrations at baseline (r = -0.17, *P* = 0.237) or post-treatment (r = -0.03, *P* = 0.821) in the entire ISN/RPS class III/IV group; similar results were seen when separate analysis was performed for the class III and IV nephritis groups (data not shown). Finally, neither baseline nor post-treatment distributions of sAxl levels were found to differ between patients with class III/IV LN in the baseline biopsy that had converted to class V in the post-treatment biopsy and patients with class III/IV LN that had converted to class I/II nephritis (*P* = 0.357 and *P* = 0.978, respectively).

Results from evaluation of the renal biopsies, albuminuria levels, creatinine concentrations, as well as eGFR and the corresponding chronic kidney disease (CKD) stages as defined by the National Kidney Foundation guidelines [38–40] are presented in Table 2. We observed moderate correlations between sAxl levels and albuminuria, both at baseline (r = 0.30, *P* = 0.030) and post-treatment (r = 0.37, *P* = 0.009). Likewise, sAxl levels correlated with creatinine levels, both at baseline (r = 0.35, *P* = 0.010) and post-treatment (r = 0.38, *P* = 0.005).

We found no correlation between sAxl levels and either renal activity or chronicity index scores at baseline (r = -0.04, *P* = 0.777 and r = 0.05, *P* = 0.726, respectively) or post-treatment (r = 0.24, *P* = 0.086 and r = 0.20, *P* = 0.166, respectively). Neither baseline nor post-treatment levels of sAxl were associated with improvements in activity index scores in renal biopsies by ≥1 (*P* = 0.301 and *P* = 0.880, respectively). Interestingly, post-treatment sAxl levels were higher in patients who worsened in renal chronicity index scores by ≥1 (median; IQR: 45.0; 32.0–55.4 ng/mL; n = 32) compared to patients who did not (median; IQR: 35.9; 27.1–44.0 ng/mL; n = 20) (*P* = 0.025); no such association was found for baseline sAxl levels (*P* = 0.110), baseline albuminuria (*P* = 0.156), or post-treatment albuminuria (*P* = 0.310).

In the pilot experiment of ISN/RPS class V glomerulonephritis (n = 12), baseline serum sAxl levels (median: 52.2; IQR: 35.2–66.2 ng/mL) were higher than sAxl levels in the healthy controls (*P*<0.001), but did not differ from levels in patients with class III/IV nephritis (*P* = 0.536), patients with class III only (*P* = 0.091) or patients with class IV only (*P* = 0.845). No significant decrease was seen following treatment (median: 46.7; IQR: 37.6–55.4; *P* = 0.814).

### Serum Axl as a biomarker of treatment response

#### Clinical response

**Fig 2:**
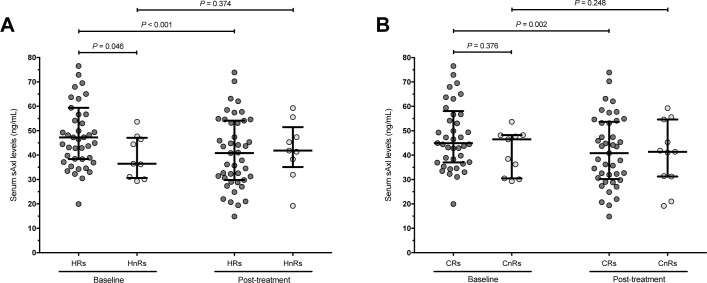
sAxl levels in relation to treatment outcomes. The scatterplots illustrate differences in soluble (s)Axl levels in patient groups stratified based on histological (A) and clinical (B) outcomes. Baseline serum sAxl levels were higher in histological responders (HRs) compared to histological non-responders (HnRs) (*P*-value derived from Mann-Whitney *U* test), and decreased following treatment in HRs but not in HnRs (*P*-values derived from Wilcoxon signed-rank tests). Accordingly, sAxl levels decreased following treatment in clinical responders (CRs) but not in clinical non-responders (CnRs). Lines and whiskers denote medians and the 25^th^ and 75^th^ percentiles. Outliers may have been omitted due to scaling.

Forty-one patients (78.8%) from the main class III/IV cohort met the criteria of clinical response (Table 3). Baseline sAxl levels did not differ between clinical responders (CRs) and clinical non-responders (CnRs) (*P* = 0.376). Following treatment, sAxl levels decreased in CRs (*P* = 0.002), but not in CnRs (*P* = 0.248) (Table 4) (Fig 2). Changes in sAxl levels expressed as ΔsAxl did not differ between CRs and CnRs (*P* = 0.583).

**Table 4:** Baseline versus post-treatment characteristics in relation to clinical response.

**Clinical responders**; n = 41	**Baseline**	**Post-treatment**	***P*-value**
SLE duration until baseline (years); M (IQR)	2.7 (0.1–8.2)	-	-
Prednisone equivalent dose (mg/day); M (IQR)	10.0 (0.0–20.0)	10.0 (7.5–15.0)	0.670
Antimalarial agents; n (%)	13 (31.7)	16 (39.0)	-
Induction treatment; n (%)			
Intravenous cyclophosphamide	29 (70.7)	-	-
Mycophenolate mofetil	9 (22.0)	-	-
Rituximab	3 (7.3)	-	-
ACE inhibitors and/or ARBs; n (%)	30 (73.2)	30 (73.2)	-
ISN/RPS class; n			
I/II	0	13	-
III (A; A/C; C)	14 (9; 5; 0)	13 (0; 6; 7)	-
IV (A; A/C; C)	27 (11; 16; 0)	5 (1; 2; 2)	-
V	0	9	-
Glomerular vasculitis	0	1	-
Activity Index; M (R)	6.0 (4.0–8.0)	1.0 (1.0–2.0)	**<0.001**
Chronicity Index; M (R)	1.0 (0.0–3.0)	2.0 (1.0–3.0)	**0.013**
Anti-dsDNA (IU/mL); M (IQR)	185.0 (39.8–600.0)	20.0 (10.8–77.3)	**<0.001**
Anti-C1q (U/mL); M (IQR)	43.1 (13.1–76.6)	12.9 (6.1–32.0)	**<0.001**
C3 (g/L); M (IQR)	0.50 (0.38–0.73)	0.82 (0.64–1.13)	**<0.001**
C4 (g/L); M (IQR)	0.07 (0.04–0.13)	0.14 (0.10–0.20)	**<0.001**
24-h U-albumin (g/day); M (IQR)	1.4 (0.6–2.5)	0.1 (0.03–0.4)	**<0.001**
Creatinine (μmol/L); M (IQR)	74.0 (60.7–94.1)	71.5 (61.6–81.4)	**0.042**
eGFR (mL/min/1.73 m^2^); M (IQR)	81.0 (68.0–98.5)	87.0 (76.5–98.0)	**0.043**
Serum sAxl levels (ng/mL); M (IQR)	44.9 (37.0–58.1)	40.8 (30.2–53.7)	**0.002**
**Clinical non-responders**; n = 11	**Baseline**	**Post-treatment**	***P*-value**
SLE duration until baseline (years); M (IQR)	2.5 (1.2–7.7)	-	-
Prednisone equivalent dose (mg/day); M (IQR)	5.0 (0.0–30.0)	12.5 (10.0–15.0)	0.759
Antimalarial agents; n (%)	2 (18.2)	1 (9.1)	-
Induction treatment; n (%)			
Intravenous cyclophosphamide	11 (100)	-	-
ACE inhibitors and/or ARBs; n (%)	8 (72.7)	8 (72.7)	-
ISN/RPS class; n			
I/II	0	2	-
III (A; A/C; C)	6 (4; 2; 0)	3 (0; 3; 0)	-
IV (A; A/C; C)	5 (5; 0; 0)	4 (1; 3; 0)	-
V	0	2	-
Activity Index; M (IQR)	7.0 (5.0–10.0)	5.0 (2.0–9.0)	0.080
Chronicity Index; M (IQR)	1.0 (0.0–1.0)	2.0 (1.0–3.0)	0.016
Anti-dsDNA (IU/mL); M (IQR)	600.0 (110.0–600.0)	45.0 (24.0–75.0)	**0.003**
Anti-C1q (U/mL); M (IQR)	63.7 (16.5–200.0)	33.7 (9.0–39.0)	**0.008**
C3 (g/L); M (IQR)	0.43 (0.30–0.77)	0.64 (0.53–0.89)	**0.014**
C4 (g/L); M (IQR)	0.10 (0.07–0.11)	0.10 (0.08–0.15)	0.100
24-h U-albumin (g/day); M (IQR)	1.9 (0.7–2.7)	1.9 (1.4–3.1)	0.755
Creatinine (μmol/L); M (IQR)	79.0 (67.7–87.5)	75.3 (56.5–83.7)	0.306
eGFR (mL/min/1.73 m^2^); M (IQR)	80.0 (74.0–90.0)	90.0 (82.0–93.0)	0.197
Serum sAxl levels (ng/mL); M (IQR)	46.5 (30.5–48.2)	41.4 (31.3–54.6)	0.248

Comparisons between baseline and post-treatment characteristics including serum soluble (s)Axl levels in patient groups stratified according to clinical response to induction treatment. The non-parametric Wilcoxon signed rank test was used for the comparisons. Statistically significant *P*-values are in bold.

ACE, angiotensin-converting enzyme; ARBs, angiotensin receptor blockers; eGFR, estimated glomerular filtration rate; h, hour; U, urine; M, median; IQR, interquartile range.

Baseline and post-treatment levels of autoantibodies are presented in Table 2. At baseline, neither anti-dsDNA nor anti-C1q levels differed between CRs and CnRs (*P* = 0.123 and *P* = 0.441, respectively). Serum anti-dsDNA levels decreased irrespective of the clinical response to treatment, both in CRs (*P*<0.001) and CnRs (*P* = 0.003). Similarly, anti-C1q levels decreased both in CRs (*P*<0.001) and CnRs (*P* = 0.008).

#### Histological response

Forty-three patients (82.7%) met the definition of histological response following treatment. Baseline serum sAxl levels were higher in HRs compared to HnRs (*P* = 0.046), and decreased following treatment in HRs (*P*<0.001) but not in HnRs (*P* = 0.374) (Table 5) (Fig 2), and high baseline levels were associated with histological response to induction therapy in multivariable logistic regression analysis after adjustment for possible confounding factors (S1 Table). Baseline sAxl levels above the 25^th^ percentile (≥36.6 ng/mL) were associated with histological response (*P* = 0.033, Fisher’s exact test), yielding a 5.5 times higher probability of response compared to lower levels in logistic regression analysis (odds ratio, OR: 5.5; coefficient: 1.7; 95% confidence interval, CI: 1.2–25.1; *P* = 0.029). This association was even more prominent after adjustment for possible confounding factors (OR: 9.3; coefficient: 2.2; 95% CI: 1.4–60.8; *P* = 0.020) (S2 Table).

**Table 5:** Baseline versus post-treatment characteristics in relation to histological response.

**Histopathological responders**; n = 43	**Baseline**	**Post-treatment**	***P*-value**
SLE duration until baseline (years); M (IQR)	2.7 (0.06–7.6)	-	-
Prednisone equivalent dose (mg/day); M (IQR)	7.5 (0.0–20.0)	10.0 (7.5–15.0)	0.907
Antimalarial agents; n (%)	14 (32.6)	17 (39.5)	-
Induction treatment; n (%)			
Intravenous cyclophosphamide	32 (74.4)	-	-
Mycophenolate mofetil	8 (18.6)	-	-
Rituximab	3 (7.0)	-	-
ACE inhibitors and/or ARBs; n (%)	32 (74.4)	32 (74.4)	-
ISN/RPS class; n			
I/II	0	16	-
III (A; A/C; C)	14 (10; 4; 0)	13 (0; 5; 8)	-
IV (A; A/C; C)	29 (14; 15; 0)	4 (1; 1; 2)	-
V	0	9	-
Glomerular vasculitis	0	1	-
Activity Index; M (R)	6.0 (4.0–8.0)	1.0 (1.0–2.0)	**<0.001**
Chronicity Index; M (R)	1.0 (0.0–2.0)	1.0 (1.0–3.0)	**0.009**
Anti-dsDNA (IU/mL); M (IQR)	185.0 (41.3–600.0)	20.5 (12.3–65.5)	
Anti-C1q (U/mL); M (IQR)	46.3 (13.3–92.8)	14.4 (5.8–36.2)	
C3 (g/L); M (IQR)	0.50 (0.38–0.73)	0.80 (0.63–1.06)	**<0.001**
C4 (g/L); M (IQR)	0.07 (0.04–0.11)	0.13 (0.10–0.18)	**<0.001**
24-h U-albumin (g/day); M (IQR)	1.4 (0.6–2.3)	0.1 (0.03–0.5)	**<0.001**
Creatinine (μmol/L); M (IQR)	72.5 (61.2–92.2)	68.0 (61.0–80.9)	**0.031**
eGFR (mL/min/1.73 m^2^); M (IQR)	84.0 (71.0–98.0)	90.0 (78.0–98.0)	**0.022**
Serum sAxl levels (ng/mL); M (IQR)	47.3 (38.4–59.4)	40.8 (29.8–54.1)	**<0.001**
**Histopathological non-responders**; n = 9	**Baseline**	**Post-treatment**	***P*-value**
SLE duration until baseline (years); M (IQR)	2.5 (1.4–11.3)	-	-
Prednisone equivalent dose (mg/day); M (IQR)	10.0 (7.5–25.0)	15.0 (11.3–15.0)	1.000
Antimalarial agents; n (%)	1 (11.1)	0 (0.0)	-
Induction treatment; n (%)			
Intravenous cyclophosphamide	8 (88.9)	-	-
Mycophenolate mofetil	1 (11.1)	-	-
ACE inhibitors and/or ARBs; n (%)	6 (66.7)	6 (66.7)	-
ISN/RPS class; n			
III (A; A/C; C)	6 (3; 3; 0)	4 (0; 4; 0)	-
IV (A; A/C; C)	3 (2; 1; 0)	5 (1; 4; 0)	-
Activity Index; M (R)	6.0 (5.0–9.0)	7.0 (5.0–9.0)	0.942
Chronicity Index; M (R)	1.0 (0.5–3.0)	3.0 (1.0–4.0)	**0.024**
Anti-dsDNA (IU/mL); M (IQR)	600.0 (115.0–600.0)	61.0 (32.5–122.0)	**0.008**
Anti-C1q (U/mL); M (IQR)	22.0 (13.1–70.1)	12.9 (9.3–36.4)	**0.038**
C3 (g/L); M (IQR)	0.60 (0.35–0.73)	0.73 (0.56–1.09)	**0.008**
C4 (g/L); M (IQR)	0.11 (0.10–0.13)	0.15 (0.09–0.17)	0.095
24-h U-albumin (g/day); M (IQR)	1.1 (0.7–2.8)	1.4 (0.4–2.2)	0.110
Creatinine (μmol/L); M (IQR)	81.9 (74.3–96.0)	81.9 (70.7–85.6)	0.678
eGFR (mL/min/1.73 m^2^); M (IQR)	78.0 (72.0–83.5)	82.0 (73.5–88.5)	0.594
Serum sAxl levels (ng/mL); M (IQR)	36.5 (30.6–47.1)	41.8 (35.1–51.5)	0.374

Comparisons between baseline and post-treatment characteristics including serum soluble (s)Axl levels in patient groups stratified according to histological response to induction treatment. The non-parametric Wilcoxon signed rank test was used for the comparisons. Statistically significant *P*-values are in bold.

ACE, angiotensin-converting enzyme; ARBs, angiotensin receptor blockers; eGFR, estimated glomerular filtration rate; h, hour; U, urine; M, median; IQR, interquartile range.

Notably, changes in sAxl levels (ΔsAxl) differed between HRs and HnRs (*P* = 0.009), with HRs showing a median of -9.4 ng/mL (corresponding to the observed decreases) and HnRs yielding a median of 1.8 ng/mL.

Neither IgG anti-dsDNA nor IgG anti-C1q levels differed between histological responders (HRs) and histological non-responders (HnRs) at baseline (*P* = 0.165 and *P* = 0.671, respectively). Similar to the pattern of decreases with regard to clinical treatment responses, anti-dsDNA levels decreased irrespective of the histological response to treatment, both in HRs (*P*<0.001) and HnRs (*P* = 0.008). In the same manner, serum anti-C1q levels decreased both in HRs (*P*<0.001) and HnRs (*P* = 0.038).

### Serum sAxl as a biomarker of the long-term renal outcome

At the 10-year follow-up, the median LM eGFR was 83.0 mL/min/1.73 m^2^ (IQR: 70.0–90.5 mL/min/1.73 m^2^) and 31/41 patients met the definition of good renal outcome while 10/41 patients did not. No assessment was available in 11 patients, either because they had not reached the 10-year follow-up by the end of the observation period (n = 9) or due to death caused by non-renal reasons (n = 2).

Using the ELNT definition of good renal outcome, baseline sAxl levels were not found to differ between patients who met the criteria at the 10-year follow-up and patients who did not (*P* = 0.643). However, post-treatment sAxl levels were lower in patients with a good long-term renal outcome (median, IQR: 37.9; 28.8–45.3 versus 46.1; 39.5–55.1 ng/mL) (*P* = 0.046). We repeated this analysis with regard to albuminuria levels; no significant difference was seen in either baseline (*P* = 0.665) or post-treatment (*P* = 0.726) albuminuria levels. The association between high post-treatment sAxl levels and non-attainment of good renal prognosis at the 10-year follow-up was still significant after adjustment for post-treatment albuminuria levels in multivariable logistic regression analysis (OR: 0.9; coefficient: -0.1; 95% CI: 0.86–0.99; *P* = 0.027) (S3 Table).

**Fig 3:**
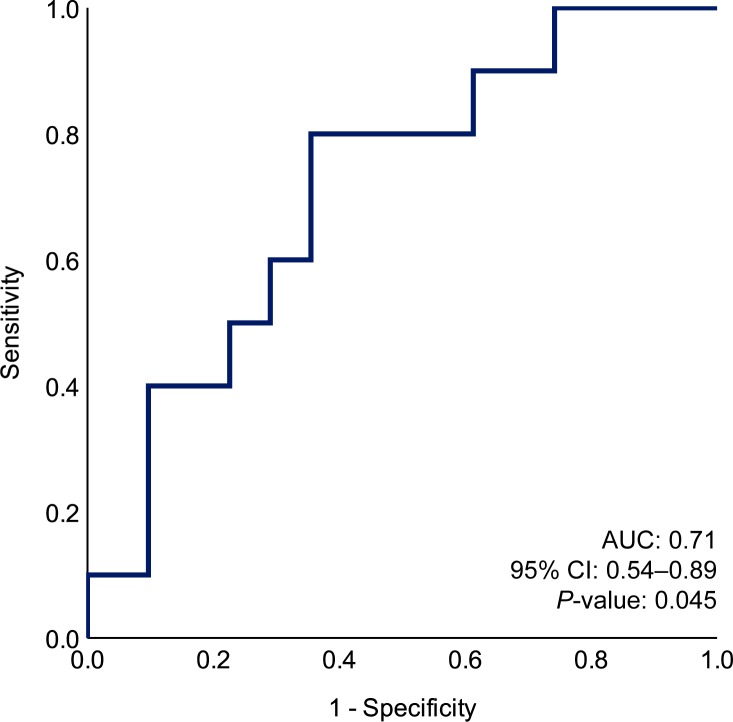
sAxl levels and long-term renal outcome. We investigated the performance of serum soluble (s)Axl levels as a predictor of unfavourable long-term renal outcome. In ROC-curve analysis, high post-treatment sAxl levels predicted non-attainment of good renal outcome at the 10-year follow-up, with the optimal threshold value being determined at a level of 41.6 ng/mL. Levels >41.6 ng/mL yielded a sensitivity of 42.1% (95% CI: 21–66%) and a specificity of 90.9% (95% CI: 69–98%) for predicting failure to attain/maintain a good renal function at 10 years from baseline. The corresponding positive predictive value (PPV, true positive) was 80.0% (95% CI: 44–96%) and the negative predictive value (NPV, true negative) was 64.5% (95% CI: 45–80%). ROC, receiver operating characteristics; AUC, area under the curve; CI, confidence interval.

We next constructed a ROC curve to illustrate post-treatment sAxl levels as a predictor of the ELNT long-term renal outcome (Fig 3). The ROC curve depicted that high post-treatment sAxl levels could predict non-attainment of good renal outcome at the 10-year follow-up (area under the curve, AUC: 0.71; 95% CI: 0.54–0.89; *P* = 0.045), and examination of the coordinates determined the optimal threshold value at a level of 41.6 ng/mL. Levels >41.6 ng/mL yielded a sensitivity of 42.1% (95% CI: 21–66%) and a specificity of 90.9% (95% CI: 69–98%) for predicting an unfavourable long-term renal outcome, with the corresponding positive predictive value (PPV, true positive) being calculated to 80.0% (95% CI: 44–96%) and the negative predictive value (NPV, true negative) to 64.5% (95% CI: 45–80%).

## Discussion

To our knowledge, this is the first longitudinal study of LN with repeat renal biopsies evaluating the performance of sAxl as a biomarker of treatment response and long-term renal outcome. We demonstrated that serum levels of soluble Axl were higher in LN patients compared to healthy individuals corroborating previous knowledge, as well as in patients with active, biopsy-proven ISN/RPS class IV versus class III LN, and they decreased following immunosuppressive treatment. Apart from the association with LN class, sAxl levels showed correlations with albuminuria and creatinine levels. Interestingly, high baseline levels predicted histology-based improvement following induction treatment and high post-treatment sAxl levels were associated with worsening in renal tissue damage and poor long-term prognosis.

Our findings add useful information to previous literature evaluating the potential role of Axl in SLE, in which sAxl levels have been demonstrated to correlate with SLE activity and autoantibody production [21, 22]. In the present study, serum levels of sAxl showed consistent associations with clinical and histological features which are coupled with renal activity in SLE patients. First, baseline serum sAxl levels were higher in patients with ISN/RPS class IV lupus nephritis compared to patients with class III glomerulonephritis, indicating that sAxl may reflect the grade of severity of renal involvement. In alignment with this, sAxl levels also correlated with albuminuria and creatinine levels both prior to and after completion of treatment. However, the lack of correlation between sAxl levels and renal activity index scores is contradictory, indicating that ISN/RPS class and renal activity index display different aspects of renal activity in patients with SLE. The decrease in sAxl levels following immunosuppression appeared to be restricted to patients with ISN/RPS class IV nephritis while no significant change was documented in patients with class III LN, presumably reflecting the initial difference in sAxl levels between the two patient groups. Importantly, the correlations with albuminuria in the light of the similar molecular weights of human sAxl and human albumin [19] implicate that the observed decreases following treatment reasonably were underestimated due to an expected decreased urinary excretion.

Interestingly, sAxl levels decreased in patients who responded to induction treatment, both according to clinical and histological evaluation, but remained stable in non-responding patients. The mechanistic explanation of these associations remains to be clarified. A first insight was recently provided by Orme et al., who documented that macrophages and B cells are a source of the soluble Axl receptor ectodomain measured in SLE sera and that the shedding of Axl inhibits the Gas6-induced anti-inflammatory signalling [20], providing evidence for an association between inflammation and disturbances to the equilibrium between membrane-bound and soluble Axl.

Further support for this notion was provided by the observation that high post-treatment sAxl levels were associated with renal damage progression. In addition, sAxl changes following induction therapy showed a response-specific pattern with reductions limited to patients who responded to treatment, unlike anti-dsDNA and anti-C1q levels which decreased irrespective of the treatment outcome. While the autoantibody response reasonably indicates an unspecific effect of immunosuppression, the stable levels of sAxl in non-responders presumably reflects a persistent state of inflammation. Accordingly, the higher post-treatment sAxl levels even in histological and clinical responders compared with the healthy individuals presumably reflect the persistent inflammatory state in patients with SLE.

Proteinuria levels have been the clinical gold standard for treatment evaluation, and low post-treatment proteinuria levels (<0.8 g/24 hours) have in recent studies been shown to predict a favourable long-term renal outcome seven years after a LN flare [36, 37, 41]. Applying the same creatinine-based criteria in the present study, proteinuria levels were not predictive of long-term renal prognosis. In contrast, we demonstrated that low post-treatment sAxl levels were associated with a favourable renal outcome at the 10-year follow-up according to the ELNT criteria, also after adjustment for levels of albuminuria. Collectively, sAxl may prove a useful marker of renal damage progression in the short term, as well as a predictor of long-term renal prognosis.

Interestingly, high baseline sAxl levels were associated with a more severe lupus nephritis class and a more impaired renal function, as well as predictive of histological response to treatment. Of course, the latter could depend on a more intense induction treatment due to the more severe clinical presentation. However, sAxl levels decreased in responding patients but not in non-responders, and high post-treatment levels were associated with progression of renal tissue damage and a more unfavourable long-term renal prognosis. Indeed, higher renal chronicity index scores in post-treatment biopsies have been shown to be associated with a poorer long-term renal outcome [6]. Together, control of renal disease activity is a necessary but not sufficient condition for the prevention of renal damage and renal function loss in the long-term; in this respect, sAxl levels may prove useful in reflecting the grade of inflammation, with high post-treatment levels indicating a need for treatment intensification.

Previous studies have suggested that Axl and Gas6 have critical roles in the development of glomerulonephritis. Mice lacking Gas6 have been shown to be protected from glomerular injury, crescent formation, and glomerular sclerosis [42]. Axl and Gas6 have also been demonstrated to be expressed by mesangial cells in glomeruli during their proliferation in a study utilising an experimental proliferative glomerulonephritis model. In this model, a soluble recombinant decoy Axl receptor was shown to inhibit mesangial cell proliferation and reduce injury by impeding the Axl/Gas6 signalling, acting as a scavenger of Gas6 [17]. In contrast, increased levels of extracellular soluble Axl have been demonstrated at a state of renal injury in human disease [23], in conformity with the findings herein. Importantly, other studies of SLE have also shown elevated levels of the ligand Gas6 at active nephritis [22]. Interestingly, soluble Axl may at least partly be in complexes together with Gas6 in the circulation [43], which may imply disturbances in the balance between functional Axl receptor and Gas6, and dysregulation of inflammatory feedback systems in chronic disease. It is worth noting that membrane-bound Axl mediates anti-inflammatory pathways, and reduced receptor levels on leukocytes abrogate important anti-inflammatory signalling. Soluble Axl may additionally act as a decoy receptor and block Gas6 interaction with other immune cells. A recent study demonstrated elevated serum levels of sAxl while membrane-bound Axl measures in peripheral blood mononuclear cell extracts were reduced in SLE patients compared to healthy controls, and showed that responsible for the cleavage of Axl from leukocytes were the cell matrix metalloproteases ADAM10 and TACE, suggesting ADAM10/TACE inhibition as a potential therapeutic modality [20]. Yet, the mechanistic manner in which the controversy between the effects of Axl seen in mesangial cells and the effects seen in immune cells are related to human disease, renal injury and lupus nephritis remains to be elucidated. In the light of a recent study showing that Axl inhibition ameliorated murine lupus-like glomerulonephritis [44], our results provide valuable information in the context of future research on Axl as a new therapeutic target in LN.

Given the known discrepancy between clinical and histological outcomes following treatment [6], a major strength of this study was the post-treatment renal biopsies, which allowed a histology-based evaluation of treatment response. Nevertheless, validation in larger LN cohorts as well as survey with a mechanistic perspective are required in order to further understand the role of Axl in renal SLE. Further survey on Axl in the membranous LN subset is also desirable. We defined clinical response based on established criteria [34], but there are no consensual response criteria for histological treatment outcomes. The histology-based assessment of treatment response used in this study and other studies by our group [35, 45] may contribute to further discussion towards the development of response criteria based on renal histology.

## Conclusions

Overall, our data suggest that sAxl levels reflect renal activity in SLE patients with proliferative glomerulonephritis, and that persistently high sAxl levels following treatment may indicate renal damage progression and a poorer long-term renal outcome. Further evaluation of serum sAxl in renal SLE is merited in order to better clarify its role, and possibly reveal a potential for modulation of the TAM pathways in future therapeutic approaches.

## Supporting information

S1 File sAxl assay protocol. The sAxl assay protocol may also be found at: dx.doi.org/10.17504/protocols.io.wnnfdde
(PDF)

S2 File Dataset. The dataset may also be found at: https://figshare.com/articles/sAxl_in_LN_-_Dataset_xlsx/7504454 doi: 10.6084/m9.figshare.7504454
(XLSX)

S1 Table Baseline sAxl levels in relation to histological response. Results from multivariable logistic regression analysis. Statistically significant P-values are in bold.
Outcome: Histological response after completion of induction therapy. Patients showing ≥50% improvement in renal activity index scores in post-treatment compared to baseline biopsies were considered histological responders; all other patients were considered histological non-responders. eGFR, estimated filtration rate; s, soluble; h, hour; U, urine; eq., equivalent; OR, odds ratio; CI, confidence interval.
(PDF)

S2 Table Baseline sAxl levels ≥36.6 ng/mL in relation to histological response. Results from multivariable logistic regression analysis. Statistically significant P-values are in bold.
Outcome: Histological response after completion of induction therapy. Patients showing ≥50% improvement in renal activity index scores in post-treatment compared to baseline biopsies were considered histological responders; all other patients were considered histological non-responders. eGFR, estimated filtration rate; s, soluble; h, hour; U, urine; eq., equivalent; OR, odds ratio; CI, confidence interval.
(PDF)

S3 Table Post-treatment sAxl levels in relation to long-term renal outcome. Results from multivariable logistic regression analysis. Statistically significant P-values are in bold.
Outcome: Good long-term renal outcome, defined as creatinine concentrations ≤88.4 μmol/L in conformity with the Euro-Lupus Nephritis Trial (ELNT). s, soluble; h, hour; U, urine; OR, odds ratio; CI, confidence interval.
(PDF)
