# Supplementary material for: Serum Axl predicts histology-based response to induction therapy and long-term renal outcome in lupus nephritis
Source: PLoS One. 2019 Feb 11;14(2):e0212068. doi: 10.1371/journal.pone.0212068 (PMC6370217; doi:10.1371/journal.pone.0212068)
Supplement: S1 File — The sAxl assay protocol may also be found at: dx.doi.org/10.17504/protocols.io.wnnfdde (PDF) [file pone.0212068.s001.pdf]

SERUM SOLUBLE (S)AXL ASSAY PROTOCOL

MATERIALS AND TOOLS

BD Vacutainer tubes (Catalogue number: 367812)

Immulon® 2 HB Flat Bottom MicroTiter® Plate

Microplate reader: ELX808 from BioTek Instruments (Winooski, VT)

Human Axl ELISA kit (Catalogue number: DY154) (R&D Systems)

- **PBS**: 137 mM NaCl, 2.7 mM KCl, 8.1 mM Na2HPO4, 1.5 mM KH2PO4, pH 7.2-7.4, 0.2 µm filtered (R&D Systems; Catalogue number: DY006).
- **Wash Buffer**: 0.05% Tween® 20 in PBS, pH 7.2-7.4 (R&D Systems®, Catalogue number: WA126).
- **Reagent Diluent**: 1% BSA (bovine serum albumin) in PBS, pH 7.2-7.4, 0.2 µm filtered (R&D Systems, Catalogue number: DY995).
- **Substrate Solution**: 1:1 mixture of Color Reagent A (H2O2) and Color Reagent B (Tetramethylbenzidine) (R&D Systems, Catalogue number: DY999).
- **Stop Solution**: 2 N H2SO4 (R&D Systems, Catalogue number: DY994).

SERUM COLLECTION AND STORAGE

1. Whole blood was collected into BD Vacutainer tubes without any additives (Catalogue number: 367812).
2. After 20 minutes of incubation at room temperature, the tubes were centrifuged 10 minutes at 3,000 rpm at room temperature.
3. The supernatant was carefully separated and aliquoted into 100 µL aliquots and stored at -80°C prior to batch processing.

ASSAY OF SERUM SAXL BY HUMAN AXL ELISA KIT (R&D SYSTEMS, MINNEAPOLIS, MINNESOTA, USA; CATALOGUE NUMBER: DY154)

1. On day one around 4pm, dilute the Capture Antibody to the working concentration in PBS without carrier protein. Immediately coat a 96-well microplate (Immulon® 2 HB flat bottom) with 50 µL per well of the diluted Capture Antibody. Seal the plate and incubate overnight at room temperature.
2. On day 2 around 8am， aspirate each well and wash with Wash Buffer three times by filling each well with Wash Buffer (300 µL) using a squirt bottle, manifold dispenser, or plate washer. Completely remove the residual liquid at the last step by tapping the inverted plate against paper towels.
3. Block plates by adding 200 µL Reagent Diluent to each well. Incubate at room temperature for 1.5 hours.
4. Prepare the samples during the blocking time. Dilute serum samples 1:50 in Reagent Diluent. Make sure to dilute enough serum samples for two wells (50 µL per well).
5. Prepare the standards during the blocking time. A seven-point standard curve using 2-fold serial dilutions in reagent diluent with the highest concentration of 4000 pg/mL is used in this assay. Make sure to make enough volume of the standards.
6. After 1.5 hours blocking, wash the plate as describe in step C2.
7. Add 50 µL of samples or standards in Reagent Diluent per well. All samples and standards should be tested in duplicate. Cover with an adhesive strip and incubate 2 hours at room temperature.
8. After 2 hours of incubation, wash the plate as describe in step C2.
9. Add 50 µL of the Detection Antibody, diluted in Reagent Diluent, to each well. Cover with a new adhesive strip and incubate 2 hours at room temperature.
10. After 2 hours of incubation, wash the plate as describe in step C2.
11. Add 50 µL of the working dilution of Streptavidin-HRP A to each well. Cover the plate and incubate for 20 minutes at room temperature. Wrap the plate with aluminum foil to avoid the plate from direct light.
12. After 20 minutes of incubation, wash the plate as describe in step C2.

13. Add 50 µL of Substrate Solution to each well. Incubate for 20 minutes at room temperature. Wrap the plate with aluminum foil to avoid the plate from direct light.
14. Add 25 µL of Stop Solution to each well. Gently tap the plate to ensure thorough mixing.
15. Determine the optical density of each well immediately, using a microplate reader set to 450 nm.
16. Calculate the Axl concentration according to the standard curve.
