# Supplementary material for: Serum Axl predicts histology-based response to induction therapy and long-term renal outcome in lupus nephritis
Source: PLoS One. 2019 Feb 11;14(2):e0212068. doi: 10.1371/journal.pone.0212068 (PMC6370217; doi:10.1371/journal.pone.0212068)
Supplement: S1 Table — Results from multivariable logistic regression analysis. Statistically significant P-values are in bold. Outcome: Histological response after completion of induction therapy. Patients showing ≥50% improvement in renal activity index scores in post-treatment compared to baseline biopsies were considered histological responders; all other patients were considered histological non-responders. eGFR, estimated filtration rate; s, soluble; h, hour; U, urine; eq., equivalent; OR, odds ratio; CI, confidence interval. (PDF) [file pone.0212068.s003.pdf]

S1 Table. Baseline sAxl levels in relation to histological response.

**Baseline variables**	**Coefficient**	**OR (95% CI)**	***P*-value**
**sAxl levels** (ng/mL)	0.11	1.12 (1.01 – 1.23)	**0.025**
**eGFR** (mL/min/1.73 m^2)	0.04	1.04 (0.99 – 1.10)	0.153
**24-h U-albumin** (g)	-0.03	0.97 (0.55 – 1.71)	0.916
**Renal Activity Index**	-0.13	0.88 (0.64 – 1.20)	0.416
**Renal Chronicity Index**	-0.21	0.81 (0.48 – 1.36)	0.424
**Prednisone eq. dose** (mg/day)	-0.05	0.96 (0.89 – 1.02)	0.183

Results from multivariable logistic regression analysis.
Statistically significant *P*-values are in bold.

Outcome: Histological response after completion of induction therapy. Patients showing ≥50% improvement in renal activity index scores in post-treatment compared to baseline biopsies were considered histological responders; all other patients were considered histological non-responders.

eGFR, estimated filtration rate; s, soluble; h, hour; U, urine; eq., equivalent; OR, odds ratio; CI, confidence interval.
