# Supplementary material for: Serum Axl predicts histology-based response to induction therapy and long-term renal outcome in lupus nephritis
Source: PLoS One. 2019 Feb 11;14(2):e0212068. doi: 10.1371/journal.pone.0212068 (PMC6370217; doi:10.1371/journal.pone.0212068)
Supplement: S2 Table — Results from multivariable logistic regression analysis. Statistically significant P-values are in bold. Outcome: Histological response after completion of induction therapy. Patients showing ≥50% improvement in renal activity index scores in post-treatment compared to baseline biopsies were considered histological responders; all other patients were considered histological non-responders. eGFR, estimated filtration rate; s, soluble; h, hour; U, urine; eq., equivalent; OR, odds ratio; CI, confidence interval. (PDF) [file pone.0212068.s004.pdf]

S2 Table. Baseline sAxl levels ≥36.6 ng/mL in relation to histological response.

**Baseline variables**	**Coefficient**	**OR (95% CI)**	***P*-value**
**sAxl levels ≥36.6 ng/mL**	2.23	9.27 (1.41 – 60.84)	**0.020**
**eGFR** (mL/min/1.73 m^2)	0.02	1.03 (0.97 – 1.08)	0.346
**24-h U-albumin** (g)	0.05	1.05 (0.57 – 1.94)	0.886
**Renal Activity Index**	-0.06	0.94 (0.71 – 1.26)	0.683
**Renal Chronicity Index**	-0.17	0.84 (0.51 – 1.37)	0.489
**Prednisone eq. dose** (mg/day)	-0.02	0.98 (0.93 – 1.04)	0.577

Results from multivariable logistic regression analysis.
Statistically significant *P*-values are in bold.

Outcome: Histological response after completion of induction therapy. Patients showing ≥50% improvement in renal activity index scores in post-treatment compared to baseline biopsies were considered histological responders; all other patients were considered histological non-responders.

eGFR, estimated filtration rate; s, soluble; h, hour; U, urine; eq., equivalent; OR, odds ratio; CI, confidence interval.
