# Supplementary material for: Serum Axl predicts histology-based response to induction therapy and long-term renal outcome in lupus nephritis
Source: PLoS One. 2019 Feb 11;14(2):e0212068. doi: 10.1371/journal.pone.0212068 (PMC6370217; doi:10.1371/journal.pone.0212068)
Supplement: S3 Table — Results from multivariable logistic regression analysis. Statistically significant P-values are in bold. Outcome: Good long-term renal outcome, defined as creatinine concentrations ≤88.4 μmol/L in conformity with the Euro-Lupus Nephritis Trial (ELNT). s, soluble; h, hour; U, urine; OR, odds ratio; CI, confidence interval. (PDF) [file pone.0212068.s005.pdf]

S3 Table. Post-treatment sAxl levels in relation to long-term renal outcome.

**Post-treatment variables**	**Coefficient**	**OR (95% CI)**	***P*-value**
**sAxl levels** (ng/mL)	-0.08	0.92 (0.86 – 0.99)	**0.027**
**24-h U-albumin** (g)	0.73	2.07 (0.66 – 6.46)	0.211

Results from multivariable logistic regression analysis.
Statistically significant *P*-values are in bold.

Outcome: Good long-term renal outcome, defined as creatinine concentrations ≤88.4 μmol/L in conformity with the Euro-Lupus Nephritis Trial (ELNT).

s, soluble; h, hour; U, urine; OR, odds ratio; CI, confidence interval.
